# Artificial Extracellular Matrices with Oversulfated Glycosaminoglycan Derivatives Promote the Differentiation of Osteoblast-Precursor Cells and Premature Osteoblasts

**DOI:** 10.1155/2014/938368

**Published:** 2014-04-28

**Authors:** Ute Hempel, Carolin Preissler, Sarah Vogel, Stephanie Möller, Vera Hintze, Jana Becher, Matthias Schnabelrauch, Martina Rauner, Lorenz C. Hofbauer, Peter Dieter

**Affiliations:** ^1^Institute of Physiological Chemistry, Faculty of Medicine Carl Gustav Carus, TU Dresden, Fiedlerstraße 42, 01307 Dresden, Germany; ^2^Biomaterials Department, INNOVENT e. V., Prüssingstraße 27 B, 07745 Jena, Germany; ^3^Max Bergmann Center of Biomaterials, TU Dresden, Budapester Straße 27, 01069 Dresden, Germany; ^4^Division of Endocrinology and Bone Diseases, Department of Medicine III, Faculty of Medicine Carl Gustav Carus, TU Dresden, Fetscherstraße 74, 01307 Dresden, Germany

## Abstract

Sulfated glycosaminoglycans (GAG) are components of the bone marrow stem cell niche and to a minor extent of mature bone tissue with important functions in regulating stem cell lineage commitment and differentiation. We anticipated that artificial extracellular matrices (aECM) composed of collagen I and synthetically oversulfated GAG derivatives affect preferentially the differentiation of osteoblast-precursor cells and *early* osteoblasts. A set of gradually sulfated chondroitin sulfate and hyaluronan derivatives was used for the preparation of aECM. All these matrices were analysed with human bone marrow stromal cells to identify the most potent aECM and to determine the influence of the degree and position of sulfate groups and the kind of disaccharide units on the osteogenic differentiation. Oversulfated GAG derivatives with a sulfate group at the C-6 position of the N-acetylglycosamine revealed the most pronounced proosteogenic effect as determined by tissue nonspecific alkaline phosphatase activity and calcium deposition. A subset of the aECM was further analysed with different primary osteoblasts and cell lines reflecting different maturation stages to test whether the effect of sulfated GAG derivatives depends on the maturation status of the cells. It was shown that the proosteogenic effect of aECM was most prominent in *early* osteoblasts.

## 1. Introduction

Extracellular matrix (ECM) is an important component of the stem cell niche influencing stem cell fate [1]. Bone marrow stromal cells (BMSC) sense not only neighbouring cells such as hematopoietic stem cells, sinusoidal endothelial cells, or fat cells but also the chemical composition of their microenvironment. In the bone marrow niche, the BMSC are in contact with several collagen types, fibronectin, and sulfated glycosaminoglycans (sGAG), mainly heparan sulfate [2]. There is growing evidence from* in vitro* studies on the importance of sGAG in facilitating the osteogenic differentiation route of BMSC [3, 4]. Heparan sulfate was identified as an important factor initiating embryonic stem cells to exit from self-renewal and regulating their lineage fate [5]. Kraushaar et al. [6] pointed out that embryonic stem cell differentiation is accompanied by structural changes of heparan sulfate, for example, by increasing degree of sulfation of N-, 3-O-, and 6-O-position. Mature bone ECM contains much less sGAG (less than 1% of bone dry weight) than the ECM of bone marrow and consists predominantly of mineralized collagen [7, 8]. Synthetically sulfated hyaluronan derivatives (sHA) and oversulfated chondroitin sulfate derivatives (sCS) as components of artificial ECM (aECM) have been recently described to promote adhesion and proliferation of dermal fibroblasts [9] and to influence osteoclastogenesis [10]. aECM with sHA derivatives are known to enhance osteogenic differentiation of hBMSC even in the absence of dexamethasone [11] which has been described as an established supplement to induce osteogenic differentiation* in vitro* [12].

In this study, a set of gradually sulfated hyaluronan and chondroitin sulfate derivatives differing in the number and the position of sulfate groups was used for the preparation of aECM. The aECM were applied as a substrate for several osteoblast precursor cells and cell lines derived from different sources and origins. We hypothesized that the response to the aECM will depend on the maturation state of the cell (line)s. TNAP activity and calcium deposition were determined as markers for osteogenic differentiation.

## 2. Materials and Methods

Unless otherwise mentioned, cell culture reagents were from Biochrom KG (Berlin Germany); fetal calf serum was from BioWest (via Th.Geyer, Hamburg, Germany); cell culture plastic ware was from Greiner BioOne (Frickenhausen, Germany) and Nunc (via Thermo Scientific, Langenselbold, Germany); and biochemical reagents were from Sigma (Taufkirchen, Germany). Rat tail collagen I was from BD Bioscience (Heidelberg, Germany) and chondroitin sulfate (sCS1;* bovine trachea)* from Sigma.

### 2.1. Preparation and Characterisation of Artificial Extracellular Matrices (aECM)

Table 1 lists the GAG derivatives which were used for the preparation of the aECM. The synthesis and characterization of (s)GAG derivatives were performed as described earlier [9, 11, 13]. The preparation of the sulfated HA derivative sHA1Δ6S was previously reported by Becher et al. [14] and Schulz et al. [15]. aECM were prepared from collagen I (col) and (s)GAG derivatives as described in [9, 11, 13]. Briefly, 1 mg collagen I was dissolved in 1 mL of ice cold 10 mM acetic acid and was mixed with an equal volume of 1 mg (s)GAG derivative/mL dissolved in ice cold double concentrated fibrillogenesis buffer (50 mM sodium dihydrogenphosphate and 11 mM potassium dihydrogenphosphate, pH 7.4). 220 *μ*L of the collagen I/(s)GAG derivative mixture per cm² was placed onto tissue culture polystyrene plates (TCPS). Fibrillogenesis was performed overnight at 37°C. The resulting aECM were air-dried, washed two times with 1 mL deionised, sterile water, and air-dried again. Before cell culture experiments aECM were rinsed with sterile PBS for 1 h at 37°C.

### 2.2. Isolation of Primary Osteoblast-Precursor Cells/Premature Osteoblasts and Cultivation of Cells

Human bone marrow stromal cells (hBMSC) were isolated from bone marrow aspirates, obtained from Caucasian donors (average age 32 ± 7 yrs., male and female donors), at the Bone Marrow Transplantation Center of the University Hospital Dresden, and were characterized as described in [16]. The donors were informed about the procedures and gave their full consent. The study was approved by the Local Ethics Commission (ethic vote No. EK114042009). Four hBMSC preparations were chosen for the experiments according to prior characterization of their osteogenic differentiation capacity and according to the characteristic “similar basal TNAP activity in passage 1.” hBMSC preparations of individual donors were not pooled. The cells were used in passages 2–4.

For isolation of primary rat osteoblasts/osteoblast-precursors, NIH guidelines for the care and use of laboratory animals were considered. Rat calvarial osteoblasts (rCaOB) were isolated from new-born Wistar rats and characterized as described [17]. Rat bone marrow stromal cells (hBMSC) were isolated from femora and tibiae of adult Wistar rats (2-3-month old). The bones were dissected and the bone marrow was flushed three times with medium containing 10% HI-FCS and antibiotics. The cell suspension was filtered through a 40 *μ*m strainer, pelleted, and treated for 4 min. with ACK solution (155 mM NH_4_Cl, 10 mM KHCO_3_, 110 mM Na_2_EDTA, pH 7.4) to remove erythrocytes. Subsequently, cells were resuspended in medium and plated onto tissue culture polystyrene (TCPS) plates. The osteoblast phenotype was confirmed by determination of tissue nonspecific alkaline phosphatase (TNAP) activity and calcium phosphate deposits. The cell lines MG-63 (CRL-1427), SaOS-2 (HTB-85), and MC3T3-E1 clone 4 (CRL-2593) were obtained from ATCC (American Tissue & Cell Culture via LGC Standards GmbH, Wesel, Germany) and cultured according to supplier's recommendations. MLO-Y4 cell line was described in detail by Kato et al. [18] and was a kind gift from Lynda Bonewald (Kansas City, USA).

Cell characteristics, plating density, and media used for cell expansion are given in Table 2. In order to have the different cell (line)s at day 4 after plating in the same state of subconfluency, hBMSC were plated with deviant cell density considering that the cells are larger in size [19]. Primary cells were used from the second to fourth passage. All media contained 2 mM glutamine and 10,000 IE penicillin/10,000 *μ*g streptomycin/mL. For the experiments, cells were plated onto TCPS or aECM-coated TCPS in medium as listed in Table 2 (=basal medium, BM). At day 4 after plating cells were cultured in BM, in BM supplemented with 10 mM *β*-glycerol phosphate and 300 *μ*M ascorbate (=osteogenic medium, OM), and in OM supplemented with 10 nM dexamethasone (dex.) (=OM/D). At day 11 after plating the activity of TNAP was determined, and at day 22 after plating the amount of deposited calcium was quantified.

### 2.3. Determination of TNAP Activity

TNAP activity was determined in cell lysates (lysis buffer: 1.5 M Tris-HCl (pH 10), 1 mM ZnCl_2_, 1 mM MgCl_2,_ and 1% Triton X-100) with p-nitrophenyl phosphate as a substrate as described previously [20]. TNAP activity was calculated from a linear calibration curve (*r* = 0.9979) prepared with p-nitrophenol. Specific TNAP activity is given in mU/mg protein. Protein concentration of the lysate was determined by Rotiquant assay (Roth GmbH, Karlsruhe, Germany) and calculated from a linear calibration curve (*r* = 0.9967) obtained with bovine serum albumin.

### 2.4. Determination of Calcium Deposition

Calcium deposition was quantified with the calcium kit (Greiner Diagnostics, Bahlingen, Germany) as previously described [11]. Cell layers were washed with PBS, dried, and incubated with 0.5 M HCl at 4°C for 24 h. Calcium content in the lysates was quantified photometrically with cresolphthalein complexone at 570 nm from a linear calibration curve (*r* = 0.9978) prepared with calcium chloride. Calcium content is given in *μ*mol/cm² culture area.

### 2.5. Morphology of hBMSC on aECM

At day 11 after plating on the different aECM, F-actin arrangement was monitored by fluorescence staining. The cells were washed gently with PBS and incubated with 4% paraformaldehyde (w/v) (Sigma, Taufkirchen, Germany) in PBS for 10 min. After permeabilization with 0.1% Triton X-100 (Sigma) in PBS for 20 min, nonspecific binding sites were blocked with 1% bovine serum albumin (w/v) (Sigma) in PBS containing 0.05% Tween-20 (Sigma). The cells were incubated with 5 U AlexaFluor-488 phalloidin/mL (Invitrogen, Karlsruhe, Germany) of PBS containing 1% bovine serum albumin and 0.05% Tween-20 at 25°C for 1 h. Subsequently 0.2 *μ*g 4′,6-diamidino-2-phenylindole (DAPI)/mL (Sigma) PBS for nuclei staining was applied for 15 min at 25°C. Afterwards, the cells were embedded in Mowiol 4–88 (Sigma) and visualized using an AxioPhot fluorescence microscope (Carl Zeiss, Oberkochen, Germany). For detection of the fluorescence, the following filters were used: excitation 450–490 nm and emission 515–565 nm for AlexaFluor-488, an excitation 365 nm and emission 420 nm for DAPI. Digital images were obtained with an AxioCam MRm camera (Carl Zeiss) by using AxioVision software release 4.6 (Carl Zeiss).

### 2.6. Statistical Analysis

Each experiment was performed with nonpooled cells from four different donors (in the case of primary cells: hBMSC, rCaOB, rBMSC) or four independent cell cultures (in the case of cell lines: MG-63, SaOS-2, MC3T3-E1, and MLO-Y4) each in triplicate. The results are presented as mean ± standard error of the mean (SEM). Statistical significance was analyzed with GraphPad Prism 5.04 software (Statcon, Witzenhausen, Germany) by one-way ANOVA (Figures 2 and 3) and two-way ANOVA analysis (Figure 4, Table 3) with Bonferroni's posttest.

## 3. Results and Discussion

### 3.1. Effect of aECM with Gradually Sulfated GAG Derivatives on TNAP Activity, Calcium Deposition, and Morphology of hBMSC

**Figure 1:**
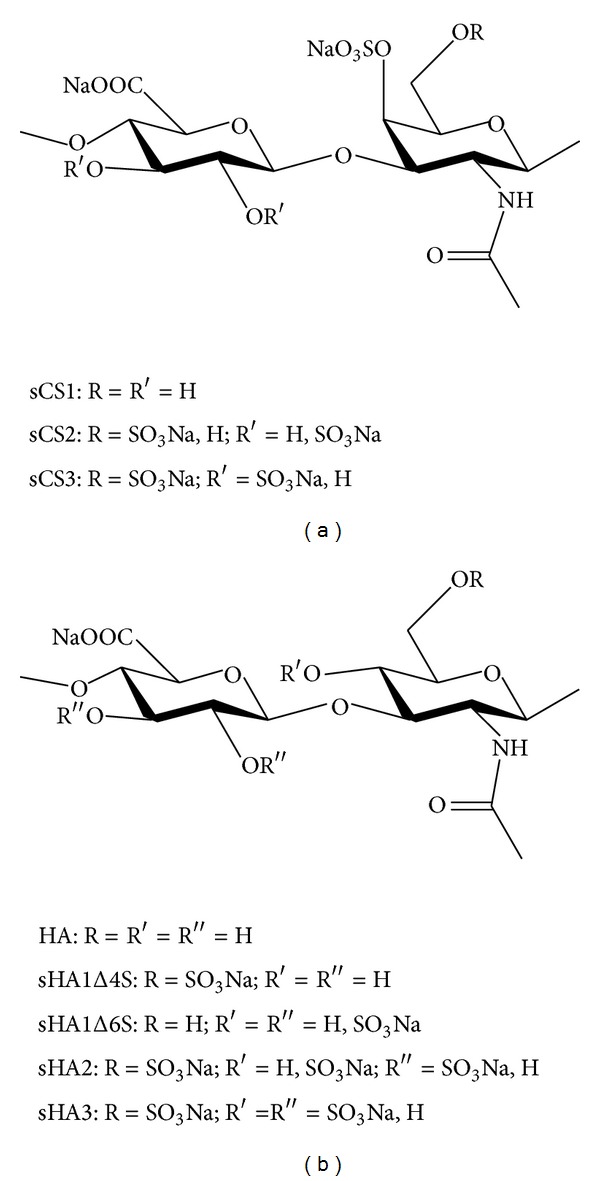
Chemical structure of sGAG derivatives. The chemical structure of chondroitin sulfate derivatives (a) and hyaluronan derivatives (b) and the most putative pattern of substituents as determined by [^13^C] nuclear magnetic resonance analysis are given.

**Figure 2:**
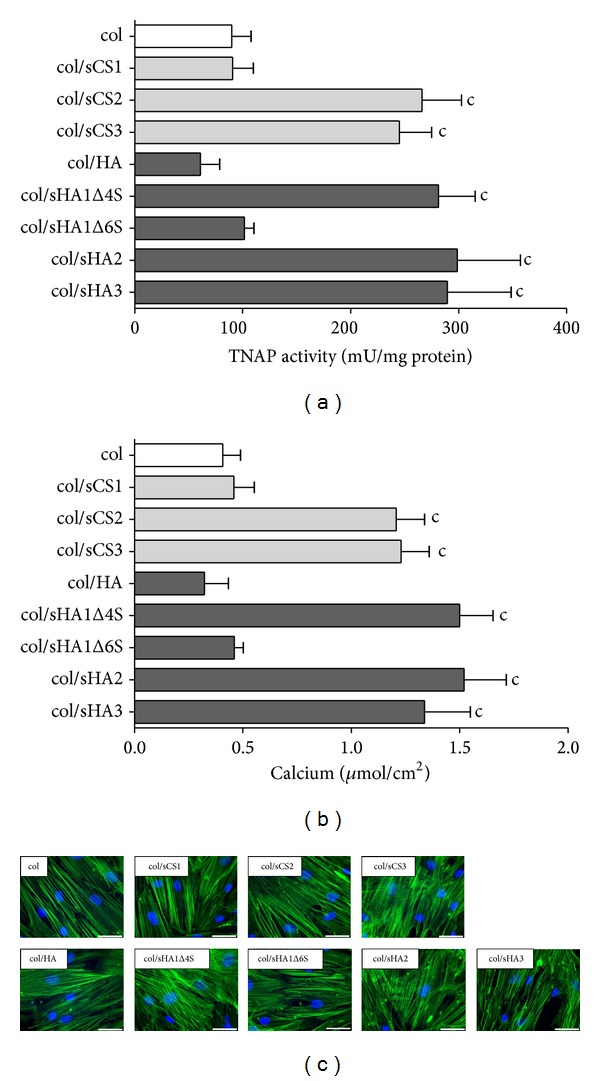
Influence of aECM on TNAP activity, calcium deposition, and morphology of hBMSC. 7,000 hBMSC were plated in BM on aECM. At day 4 after plating, BM was replaced with OM/D. At day 11 after plating, TNAP activity was determined in cell lysates with p-nitrophenylphosphate as a substrate (a). The released p-nitrophenolate was measured photometrically at 405 nm. TNAP activity in mU/mL was normalized to protein concentration in mg/mL determined with Rotiquant assay. At day 22 after plating, calcium deposition was quantified with cresolphthalein complexone at 570 nm (b). Significant differences of col/sGAG-aECM versus col-aECM were calculated by one-way ANOVA analysis and indicated with c (*P* < 0.001), *n* = 4. (c) At day 11 after plating, cells were stained for F-actin fibres with Alexa488-phallodin (green fluorescence) and for nuclei with DAPI (blue fluorescence), scale bar = 50 *μ*m.

Gradually sulfated GAG derivatives were synthesized using natural chondroitin sulfate (C4S = CSA = sCS1) (Figure 1(a)) and natural hyaluronan (Figure 1(b)) as educts. These derivatives were used to prepare collagen I matrices with about 2–7% (w/w) of GAG (for characteristics see [11, 13]). Using aECM with gradually sulfated GAG derivatives as a substrate for hBMSC, we addressed the effects of the degree of sulfation, the position of sulfate group(s), and the kind of disaccharide unit (HA: glucuronic acid (GlcA)-N-acetyl-glucosamine (NAcGlc); CS: GlcA-N-acetyl-galactosamine (NAc-Gal)) on TNAP activity and calcium deposition. aECM composed of col were used as a reference. hBMSC were plated in BM onto aECM, differentiated in OM/D from day 4, and analysed at day 11 after plating for TNAP activity and at day 22 after plating for calcium deposition. Both parameters were determined as indicators of osteogenic differentiation [12]. aECM containing sCS2, sCS3, sHA1Δ4S, sHA2, and sHA3 caused a significant increase of TNAP activity by about threefold in comparison to col-aECM (Figure 2(a)) whereas aECM containing sCS1, hyaluronan, and sHA1Δ6S did not alter TNAP activity. Once sufficient phosphate was released by TNAP from the* in vitro*-substrate *β*-glycerol phosphate (component of OM/D) the mineralisation took place. The elevated calcium deposition around hBMSC on aECM containing sCS2, sCS3, sHA1Δ4S, sHA2, and sHA3 (Figure 2(b)) correlated with increased TNAP activity. The different aECM did not cause appreciable changes of cell morphology as assessed by F-actin staining (Figure 2(c)). A well-organized, dense F-actin cytoskeleton forming strong fibres was seen in hBMSC on col, col/sCS1, col/HA, col/sHA1Δ4S, and col/sHA1Δ6S. On col/sCS2, col/sCS2, col/sHA2, and col/sHA3, the F-actin fibres looked slightly tender; nevertheless, they formed also a tight cytoskeletal network.

Sulfated GAG derivatives* per se* and as a component of aECM were shown to enhance osteogenic differentiation of hBMSC [11, 20]; however, it was not really clear whether the effect was dependent on the number and/or position of sulfate groups and was influenced by the kind of disaccharide unit. Here we demonstrated with hBMSC that the effect of col/sGAG-aECM on TNAP activity and calcium deposition was independent of the kind of disaccharide units (GlcA-GlcNac or GlcA-GalNAc) and the absolute number of sulfated groups. This effect was seen to the same extent with sulfated chondroitin sulfate derivatives sCS2 and sCS3 and all sulfated hyaluronan derivatives (besides sHA1Δ6S), respectively. An increasing degree of sulfation (sCS3 versus sCS2; sHA2 and sHA3 versus sHA1Δ4S) did not significantly alter the effect on TNAP activity and calcium deposition. Other than the number of sulfate groups their position seems to be important: sGAG derivatives without sulfate group in C-6 position but with a sulfate group at C-4 position of the N-acetyl-glucosamine such as the native chondroitin-4-sulfate (sCS1) and the sHA1Δ6S derivative did not increase TNAP activity and calcium deposition, suggesting that the sulfation of the hydroxyl group at C-6 position of the N-acetyl-glucosamine is necessary for the proosteogenic effect of sGAG. For a further proof of this hypothesis, the use of chondroitin-6-sulfate as a component of aECM would be helpful. Sufficiently pure chondroitin-6-sulfate, however, was not available; the commercially chondroitin-6-sulfate consists to more than the half of chondroitin-4-sulfate [11].

### 3.2. Influence of aECM on Osteoblast-Precursor Cells, Premature and Mature Osteoblasts

**Figure 3:**
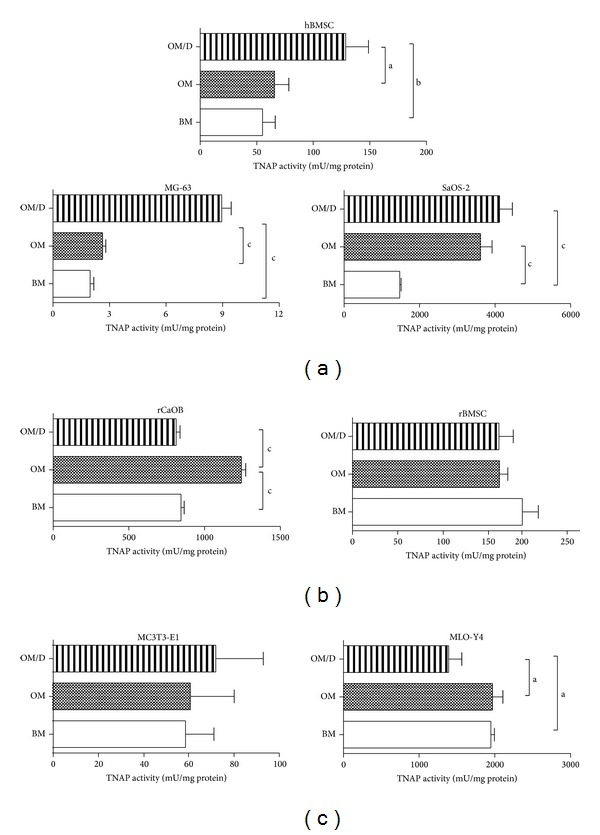
Influence of medium supplements on TNAP activity of osteoblast-precursor cells, premature and mature osteoblasts. Various human (hBMSC, MG-63, SaOS-2) (a), rat (rCaOB, rBMSC) (b), and mouse cell (line)s (MC3T3-E1, MLO-Y4) (c) were plated in BM on TCPS. At day 4 after plating cells were cultured either in BM, OM, or OM/D. At day 11 after plating, TNAP activity was determined in cell lysates with p-nitrophenylphosphate as a substrate. The released p-nitrophenolate was measured photometrically at 405 nm. TNAP activity in mU/mL was normalized to protein concentration in mg/mL determined with Rotiquant assay. Significant differences were calculated by one-way ANOVA analysis and indicated with a (*P* < 0.05), b (*P* < 0.01) and c (*P* < 0.001), *n* = 4.

Various primary cells and cell lines of human, rat, and mouse origin were used to investigate whether the effect of aECM on osteogenic differentiation is restricted solely to human BMSC or whether it is obtained also for cells of other species. To see if the response to the aECM depends on the maturation stage of the cells, osteoblast-precursor cells, premature and mature osteoblasts of human, rat and mouse origin were subjected to col/sCS3- and col/sHA3-aECM in comparison to col-, col/sCS1-, and col/HA-aECM. The* in vitro* experiments were performed with human and rat bone marrow-derived stromal cells (BMSC) reflecting osteoblast-precursor cells, with human MG-63 cell line, primary rat calvaria osteoblasts (rCaOB) and mouse MC3T3-E1 cell line reflecting* early* premature osteoblasts, and with human SaOS-2 cell line and mouse MLO-Y4 cell line reflecting* late* mature osteoblasts, respectively. To evaluate whether *β*-glycerol phosphate and ascorbate are sufficient supplements for the induction of osteogenic differentiation or whether dexamethasone is mandatory, the cells were cultured on TCPS in basal medium (BM), osteogenic medium (OM) containing *β*-glycerol phosphate and ascorbate, and OM/D (OM supplemented with dexamethasone), respectively, and analysed for TNAP activity at day 11 (Figure 3). Dexamethasone (OM/D) was essential to induce TNAP activity in both human BMSC and MG-63 (Figure 3(a)) but not in rBMSC and rCaOB (Figure 3(b)). *β*-Glycerol phosphate and ascorbate (OM) were sufficient for human SaOS-2 to induce TNAP activity; with OM/D no further increase of TNAP activity was seen (Figure 3(a)). In rCaOB, an increase of TNAP activity was seen with OM, whereas in the presence of dexamethasone TNAP remained at the level of BM (Figure 3(b)). TNAP activity of rBMSC (Figure 3(b)) and MC3T3-E1 (Figure 3(c)) was not dependent on medium supplements. In MLO-Y4, dexamethasone caused a significant decrease of TNAP activity (Figure 3(c)).

**Figure 4:**
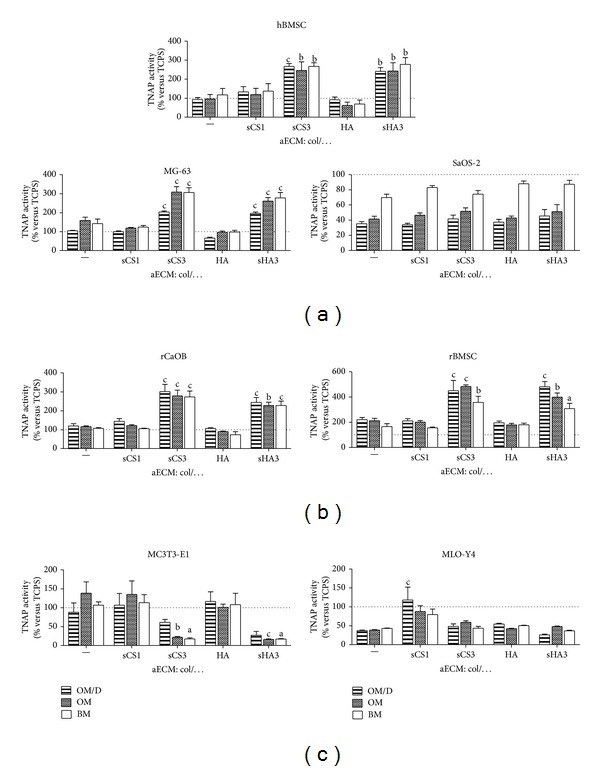
Influence of aECM on TNAP activity of osteoblast-precursor cells, premature and mature osteoblasts. Various human (hBMSC, MG-63, SaOS-2) (a), rat (rCaOB, rBMSC) (b), and mouse cell (line)s (MC3T3-E1, MLO-Y4) (c) were plated in BM on aECM composed of collagen I and sCS1, sCS3, HA, and sHA3, respectively. At day 4 after plating cells were cultured either in BM, OM, or OM/D. At day 11 after plating, TNAP activity was determined in cell lysates with p-nitrophenylphosphate as a substrate. The released p-nitrophenolate was measured photometrically at 405 nm. TNAP activity in mU/mL was normalized to protein concentration in mg/mL determined with Rotiquant assay. Data are presented as % of TCPS values in the corresponding cell culture medium. Significant differences of OM/D, OM, and BM on (s) GAG versus col-aECM were calculated by two-way ANOVA and indicated with a (*P* < 0.05), b (*P* < 0.01), and c (*P* < 0.001), *n* = 4.

Cells were plated on col-aECM as a reference and on col-aECM containing sCS1, sCS3, HA, and sHA3. They were cultured in BM, OM, and OM/D and analysed for TNAP activity at day 11 (Figure 4). aECM with sCS3 and sHA3 induced an increase of TNAP activity in hBMSC, MG-63, rCaOB, and rBMSC; this effect was not dependent on the addition of osteogenic supplements and always present in BM (Figures 4(a) and 4(b)). TNAP activity of SaOS-2 was rather negatively influenced by all aECM compared to TCPS (dotted line = 100%; Figure 4(a)); in comparison to BM, the effect was more pronounced when SaOS-2 were cultured in OM/D and OM. In MC3T3-E1, the contact to sCS3- and sHA3-containing aECM caused a decrease of TNAP activity; the other aECM did not alter TNAP activity (Figure 4(c)). TNAP activity of MLO-Y4 was higher in cells on col/sCS1-aECM than in cells on col-aECM (Figure 4(c)).

The results of calcium determination on aECM at day 22 are summarized in Table 3. In all cell (line)s, a marked calcium deposition was only seen when the cells were cultured in OM and OM/D (both containing *β*-glycerol phosphate). In comparison to col-aECM, aECM with sCS3 and sHA3 induced a significant increase of calcium deposition in hBMSC, MG-63, rCaOB, and rBMSC. The decrease of calcium deposition in MC3T3-E1 on col/sCS3- and col/sHA3-aECM correlated to the effect of these aECM on TNAP activity. With SaOS-2 cells no significant differences in calcium deposition between the different aECM were found in OM/D and OM. According to the effect on TNAP activity, col/sCS1-aECM caused in MLO-Y4 cells in OM/D a significant higher calcium deposition in comparison to col-aECM.

The results indicate that primary osteoblasts-precursor cells as well as premature osteoblasts (BMSC, MG-63, and* early* osteoblasts isolated from calvariae of newborn rats) are responsive to aECM with oversulfated GAG derivatives such as col/sCS3 and col/sHA3. BMSC are multipotent precursor cells which can be differentiated into fibrogenic, myogenic, neuronal, osteogenic, chondrogenic, and adipogenic lineage; the signals driving them into a particular differentiation route come from intrinsic genetic programming, diverse soluble mediators, and the ECM. [21, 22]. Both MG-63 and SaOS-2 are human osteosarcoma-derived cell lines; MG-63 cells are referred to as premature osteoblasts; SaOS-2 cells are characterized as mature osteoblasts [23–26]. In contrast to MG-63, SaOS-2 cells did not respond to aECM with high-sulfated GAG derivatives; no differences in TNAP activity and calcium deposition were determined on all used aECM. TNAP activity and calcium deposition of SaOS-2 was increased with all aECM in the presence of osteogenic supplements (OM and OM/D). Both mouse MC3T3-E1 which are premature osteoblasts and MLO-Y4 which are senescent osteoblasts did not response on aECM with high-sulfated GAG derivatives. For MC3T3-E1 it was reported that a natural oversulfated chondroitin sulfate derivative, isolated from squid cartilage and consisting of GlcA 1→3 GalNAc (C4-sulfate and C6-sulfate) disaccharide units, enhanced the deposition of collagen and calcium phosphate [27]. In contrast, our studies showed that aECM with high-sulfated GAG derivatives (col/sCS3, col/sHA3) caused in MC3T3-E1 a decrease of TNAP activity compared to col-aECM. Reasonably this cell line share some but not all of features of primary osteoblasts [28]. The osteocyte cell line MLO-Y4 responded in OM/D—unlike the other osteoblasts—to col/sCS1 with a significant increase of TNAP activity and calcium deposition compared to col-aECM. MLO-Y4 cells reflect the most mature* late* osteoblast phenotype used in this study [29, 30].* Late* osteoblasts/osteocytes derive from active osteoblasts which had synthesized new bone matrix and become incorporated therein [31]. Bone matrix maturation is associated with altered GAG composition switching from heparan sulfate-proteoglycans which are mainly responsible for interaction with mediator proteins to chondroitin sulfate-proteoglycans which are less potent in mediator binding but support calcium accumulation [32, 33]. In adult bone matrix, chondroitin-4-sulfate (=sCS1) was seen to be the most abundant GAG [34]. The results of this study suggest that synthetically sulfated GAG derivatives could partially reflect the bone marrow environment and its influence on early osteogenic differentiation.

## 4. Conclusion

Artificial ECM with collagen I and oversulfated GAG derivatives provide a cellular microenvironment which facilitates the osteogenic differentiation preferentially of osteoblast-precursor cells and* early* osteoblasts. The sulfate group in C-6 position of the N-acetyl- glucosamine seems to be mandatory for the proosteogenic effect of sulfated GAG derivatives. Osteoblast-precursor cells and* early* osteoblasts as hBMSC and MG-63 revealed a pronounced osteogenic differentiation on aECM with oversulfated GAG derivatives even in the absence of dexamethasone. The strong osteoinductive effect of aECM with oversulfated GAG derivatives (partially independent on dexamethasone) makes them an interesting tool for tissue engineering approaches. They are a structurally well-characterised alternative to natural GAG to address the effects of sulfated GAG on* early* osteogenic differentiation to a defined GAG structure.

## Figures and Tables

**Table 1:** Characteristics of the GAG derivatives.

Sample		DS_S_^a^	M_W_^b^ [g/mol]	PD^c^
sCS1	Chondroitin sulfate	1	17,900	1.4
sCS2	Sulfated chondroitin sulfate	2	23,200	1.5
sCS3	Sulfated chondroitin sulfate	3	19,900	1.5

HA	Hyaluronan	0	1,17 × 10^6^	4.8
sHA1Δ4S	Sulfated hyaluronan	1	26,400	2.0
sHA1Δ6S	Sulfated hyaluronan	1	42,600	2.0
sHA2	Sulfated hyaluronan	2	26,400	1.8
sHA3	Sulfated hyaluronan	3	47,800	1.7

^a^DS_S_: degree of sulfation (average number of sulfate groups per disaccharide repeating unit).

^b^M_W_: weight-average molecular weight determined by gel permeation chromatography (GPC) using laser light scattering (LLS) detection.

^c^PD: polydispersity index, determined from the molecular weight distribution calculated from the number-average and weight average molecular weights obtained by GPC with refraction index (RI) detection.

**Table 2:** Cell culture conditions.

Cells	Species	Expansion medium	Seeding density [cells/cm²]	Source	Characteristics	
hBMSC	Human	DMEM/10% HI-FCS	7,000	Iliac crest/bone marrow	Primary cells	Osteoblast-precursors
MG-63	Human	DMEM/10% HI-FCS	12,500	Long bones/osteosarcoma	Cell line	Premature osteoblasts
SaOS-2	Human	McCoys5A/15% HI-FCS	12,500	Long bones/osteosarcoma	Cell line	Mature osteoblasts

rCaOB	Rat	DMEM/10% HI-FCS	12,500	Skull/calvariae	Primary cells	Premature osteoblasts
rBMSC	Rat	DMEM/10% HI-FCS	12,500	Long bones/bone marrow	Primary cells	Osteoblast-precursors

MC3T3-E1	Mouse	*α*-MEM/10% HI-FCS	12,500	Skull/calvariae	Cell line	Premature osteoblasts
MLO-Y4	Mouse	*α*-MEM/10% HI-FCS	12,500	Long bones/femora, tibiae	Cell line	Senescent osteoblasts (osteocytes)

**Table 3:** Influence of aECM on mineralisation of osteoblast-precursor cells, premature and mature osteoblasts.

		aECM				
		col	col/sCS1	col/sCS3	col/HA	col/sHA3
hBMSC	OM/D	0.540 ± 0.066	0.778 ± 0.164	1.563 ± 0.086^c^	0.538 ± 0.082	1.405 ± 0.116^b^
	OM	0.286 ± 0.068	0.355 ± 0.098	0.734 ± 0.135^b^	0.186 ± 0.049	0.724 ± 0.130^b^
	BM	0.093 ± 0.004	0.044 ± 0.007	0.070 ± 0.007	0.074 ± 0.002	0.096 ± 0.009
MG-63	OM/D	0.043 ± 0.001	0.040 ± 0.002	0.083 ± 0.002^c^	0.027 ± 0.002	0.080 ± 0.003^c^
	OM	0.019 ± 0.002	0.014 ± 0.001	0.037 ± 0.003^c^	0.012 ± 0.001	0.031 ± 0.002^c^
	BM	0.003 ± 0.002	0.001 ± 0.001	0.007 ± 0.002	0.009 ± 0.001	0.005 ± 0.003
SaOS-2	OM/D	6.584 ± 0.510	6.360 ± 0.322	7.766 ± 0.964	6.982 ± 0.689	8.495 ± 1.550
	OM	6.865 ± 0.639	7.652 ± 0.519	8.504 ± 0.727	7.059 ± 0.393	8.466 ± 1.515
	BM	0.058 ± 0.010	0.050 ± 0.058	0.059 ± 0.029	0.068 ± 0.055	0.035 ± 0.011

rCaOB	OM/D	4.408 ± 0.439	5.323 ± 0.526	11.09 ± 1.401^c^	3.937 ± 0.123	8.995 ± 0.961^c^
	OM	6.552 ± 0.230	6.883 ± 0.244	15.70 ± 1.730^c^	5.135 ± 0.122	12.86 ± 0.981^b^
	BM	0.075 ± 0.079	0.027 ± 0.006	0.048 ± 0.022	0.003 ± 0.005	0.014 ± 0.003
rBMSC	OM/D	1.625 ± 0.122	1.542 ± 0.129	3.296 ± 0.598^c^	1.444 ± 0.104	3.521 ± 0.311^c^
	OM	1.559 ± 0.138	1.478 ± 0.086	3.553 ± 0.087^c^	1.325 ± 0.088	2.931 ± 0.240^b^
	BM	0.009 ± 0.002	0.017 ± 0.005	0.030 ± 0.020	0.021 ± 0.010	0.010 ± 0.002

MC3T3-E1	OM/D	0.288 ± 0.080	0.352 ± 0.101	0.200 ± 0.026^a^	0.384 ± 0.082	0.090 ± 0.031^b^
	OM	0.384 ± 0.082	0.376 ± 0.098	0.062 ± 0.005^b^	0.279 ± 0022	0.044 ± 0.003^c^
	BM	0.005 ± 0.002	0.011 ± 0.005	0.006 ± 0.002	0.008 ± 0.003	0.005 ± 0.001
MLO-Y4	OM/D	2.359 ± 0.064	7.462 ± 2.161^c^	3.045 ± 0.423	3.436 ± 0.126	1.633 ± 0.132
	OM	3.436 ± 0.126	7.849 ± 1.348	5.271 ± 0.371	3.783 ± 0.108	4.271 ± 0.108
	BM	0.084 ± 0.006	0.028 ± 0.007	0.050 ± 0.014	0.048 ± 0.009	0.009 ± 0.007

Calcium amount [*μ*mol/cm²] given as mean ± SEM; significant differences of OM/D, OM, and BM on (s) GAG versus col-aECM were calculated by two-way ANOVA and indicated with ^a^(*P* < 0.05), ^b^(*P* < 0.01), and ^c^(*P* < 0.001), *n* = 4.

## Abbreviations

(a)ECM: (Artificial) Extracellular matrix
*α*-MEM: Minimal essential medium
CS: Chondroitin sulfate
Dex: Dexamethasone
DMEM: Dulbecco's minimal essential medium
DS_S_: Degree of sulfation per disaccharide repeating unit
HA: Hyaluronan
h/rBMSC: Human/rat bone marrow stromal cells
HI-FCS: Heat-inactivated fetal calf serum
PBS: Phosphate-buffered saline
rCaOB: Rat calvaria osteoblast
sCS1: Low-sulfated CS = chondroitin-4-sulfate = chondroitin sulfate A (DS_S_ ~ 1)
sCS2: Medium-sulfated CS derivate (DS_S_~2)
sCS3: High-sulfated CS derivate (DS_S_~3)
(s)GAG: (Sulfated) Glycosaminoglycan
sHA1Δ4S and sHA1Δ6S: Low-sulfated HA derivatives (DS_S_~1)
sHA2: Medium-sulfated HA derivative (DS_S_~2)
sHA3: High-sulfated HA derivative (DS_S_~3)
TCPS: Tissue culture polystyrene
TNAP: Tissue nonspecific alkaline phosphatase.
